# Fc gamma receptor-TLR cross-talk elicits pro-inflammatory cytokine production by human M2 macrophages

**DOI:** 10.1038/ncomms6444

**Published:** 2014-11-13

**Authors:** Lisa T. C. Vogelpoel, Ivo S. Hansen, Theo Rispens, Femke J. M. Muller, Toni M. M. van Capel, Maureen C. Turina, Joost B. Vos, Dominique L. P. Baeten, Martien L. Kapsenberg, Esther C. de Jong, Jeroen den Dunnen

**Affiliations:** 1 Department of Cell Biology and Histology, Academic Medical Center, University of Amsterdam, 1105 AZ Amsterdam, The Netherlands; 2 Department of Immunopathology, Sanquin Research and Landsteiner Laboratory, Academic Medical Center, University of Amsterdam, 1006 AN Amsterdam, The Netherlands; 3 Department of Clinical Immunology and Rheumatology, Academic Medical Center, University of Amsterdam, 1105 AZ Amsterdam, The Netherlands; 4 Immunaffect B.V., 1404 AK Bussum, The Netherlands; 5 Department of Molecular Cell Biology and Immunology, Free University Medical Center, 1007 MB Amsterdam, The Netherlands

## Abstract

M2 macrophages suppress inflammation in numerous disorders, including tumour formation, infection and obesity. However, the exact role of M2 macrophages in the context of several other diseases is still largely undefined. We here show that human M2 macrophages promote inflammation instead of suppressing inflammation on simultaneous exposure to complexed IgG (c-IgG) and TLR ligands, as occurs in the context of diseases such as rheumatoid arthritis (RA). c-IgG-TLR ligand co-stimulation of M2 macrophages selectively amplifies production of pro-inflammatory cytokines TNF-α, IL-1β and IL-6 and promotes Th17 responses, which all play a critical role in RA pathology. Induction of pro-inflammatory cytokines on c-IgG co-stimulation mainly depends on Fc gamma receptor IIa (FcγRIIa), which selectively amplifies cytokine gene transcription and induces caspase-1 activation. These data indicate that FcγR-TLR cross-talk may be targeted for treatment to attenuate inflammation in RA, by restoring the anti-inflammatory function of M2 macrophages.

## Abstract (web summary)

M2-polarized macrophages are generally considered anti-inflammatory, but whether polarization markers always reflect functional states remains debatable. Here the authors show that complexed IgG and TLR co-stimulation, observed in infections or rheumatoid arthritis, elicits an inflammatory response in M2 macrophages.

## Graphical abstract

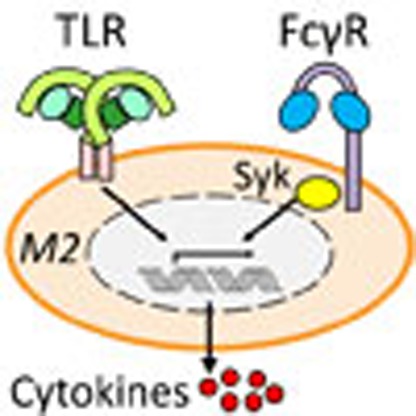

Macrophages are a heterogeneous population of myeloid cells that can differentiate into a full spectrum of different phenotypes, which coexist within tissues^1^. In a simplified model of macrophage polarization, classically activated macrophages with pro-inflammatory properties are referred to as M1 macrophages, whereas macrophages with anti-inflammatory, regulatory and/or wound-healing properties are called M2 macrophages^2^,^3^. Indeed, M2 macrophages have been described to suppress inflammation in numerous disorders, including tumour formation, infection, atherosclerosis and obesity^1^. However, the role of M2 macrophages in others disorders, including rheumatoid arthritis (RA), is still unclear^1^,^4^.

RA is a complex autoimmune disease characterized by chronic joint inflammation that affects 1% of the population, of which the exact cause is still unknown^5^,^6^,^7^. Macrophages are considered to be central players in RA pathogenesis, since they are the main producers of pro-inflammatory cytokines that promote inflammation^4^,^5^,^6^,^7^. Predominant cytokines in RA pathogenesis are tumour-necrosis factor (TNF-α), IL-1β and IL-6, which act both locally and systemically and are currently a main target for RA therapy^5^,^6^,^7^. Although classification of M1 and M2 macrophage subsets in humans is not as clear as in mice^3^, both subsets can be identified in inflamed synovia of RA patients^8^,^9^,^10^,^11^, using haemoglobin scavenger receptor CD163 as the main marker for human M2 macrophages, both *in vitro* and *in situ*^2^,^12^.

On the basis of the anti-inflammatory properties of M2 macrophages in aforementioned disorders, M2 macrophages have been suspected to suppress inflammation in RA^1^,^4^. However, exploration of functional consequences of macrophage polarization in RA has just commenced, and little is known about the exact role of M2 macrophages in this disease^4^. Therefore, in this study we set out to investigate how human M2 macrophages respond in the context of RA. Strikingly, we show that instead of suppressing inflammation, exposure of M2 macrophages to RA-associated agonists strongly promotes inflammation.

## Results

### Pro-inflammatory cytokine production by M2 macrophages

To study the function of human M2 macrophages in the context of RA, we compared the response of M2 with M1 macrophages on exposure to RA-associated agonists. The most commonly used method for generating human *in vitro* M1 and M2 macrophages is differentiating macrophages from monocytes using either granulocyte–macrophage colony-stimulating factor (GM-CSF) or M-CSF, respectively^13^,^14^,^15^,^16^. Phenotypical analysis indeed confirmed that M-CSF-differentiated M2 macrophages displayed a classical M2 phenotype compared with GM-CSF M1 macrophages, characterized by high CD163 and CD14 protein expression^12^,^16^,^17^ and *SLC40A1*, *FOLR2* and *HMOX1* mRNA expression^18^,^19^ (Supplementary Fig. 1a,b).

**Figure 1:**
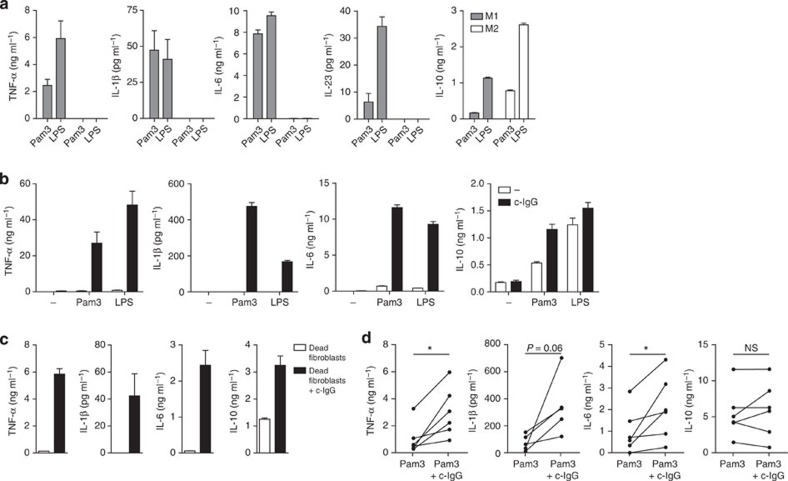
c-IgG-TLR ligand co-stimulation of M2 macrophages induces pro-inflammatory cytokines. (**a**) M1 or M2 macrophages were stimulated with Pam3CSK4 (Pam3) or LPS. (**b**) M2 macrophages were stimulated with TLR ligands, c-IgG or a combination. (**c**) M2 macrophages were stimulated with dead synovial fibroblasts either combined with c-IgG or not. (**a**–**c**) After 24 h, cytokine levels were determined by ELISA, mean+s.e.m. Data are representative of at least (**a**,**b**) twenty or (**c**) three experiments, performed in triplicate, with different donors. (**d**) CD163^+^ cells were isolated from synovial fluid of active RA patients (*n*=6) and stimulated with Pam3CSK4 or Pam3CSK4 combined with c-IgG. After 24 h, cytokine levels were determined by ELISA. Each pair of dots represents one patient. *P≪0.05; NS, not significant, two-tailed Wilcoxon matched-pair test.

An increasing body of evidence indicates that macrophage activation in RA patients strongly depends on the family of Toll-like receptors (TLRs). Most likely, TLR-induced macrophage activation occurs through recognition of endogenous ligands, also referred to as damage-associated molecular patterns, which are abundantly present in RA synovia as a result of extensive tissue damage and cell death^20^,^21^. To assess the consequences of TLR activation, we stimulated macrophages with TLR ligands Pam3CSK4 and LPS, which specifically activate TLR2 and TLR4, respectively. As expected, TLR stimulation of M1 macrophages induced the production of numerous pro-inflammatory cytokines including TNF-α, IL-1β, IL-6, IL-23 and limited amounts of IL-10 (Fig. 1a). In contrast, stimulation of M2 macrophages hardly induced any production of pro-inflammatory cytokines, but did induce production of anti-inflammatory IL-10 (Fig. 1a), thereby confirming the anti-inflammatory response of M2 macrophages on exposure to TLR ligands.

Besides TLR ligands, also autoantibodies have been implicated in the pathogenesis of RA^6^,^22^. The most specific autoantibodies for RA, that is, anti-citrullinated protein antibodies (ACPA), are mainly of the IgG isotype^23^. In RA synovia, ACPA form IgG immune complexes by recognition of citrullinated proteins, which are mostly present in insoluble amorphous deposits^24^. To mimic these insoluble IgG immune complexes, we used complexed IgG (c-IgG), that is, purified human IgG coated on high-affinity plates. As shown in Fig. 1b, individual stimulation of M2 macrophages with c-IgG induced little amounts of cytokines. However, in inflamed RA synovia, macrophages are simultaneously exposed to both c-IgG and TLR ligands. Strikingly, co-stimulation of c-IgG with TLR ligands strongly induced the production of pro-inflammatory cytokines TNF-α, IL-1β and IL-6 by M2 macrophages (Fig. 1b). IL-10 production was upregulated as well, albeit moderately compared with the pro-inflammatory cytokines (Fig. 1b). To verify that this response is also induced on exposure to damage-associated molecular patterns that are relevant in RA, we stimulated M2 macrophages with damaged and dead (freeze–thawed) human synovial fibroblasts, in combination with c-IgG. Similar to stimulation with specific microbial TLR ligands, co-stimulation of M2 macrophages with c-IgG also synergistically increased production of TNF-α, IL-1β and IL-6 on exposure to dead synovial fibroblasts, while IL-10 was only moderately affected (Fig. 1c).

c-IgG co-stimulation also upregulated cytokine production by M1 macrophages, although the relative effect was far more pronounced in M2 macrophages, either using Pam3CSK4 or LPS (Supplementary Fig. 2a,b). To assess whether c-IgG-TLR ligand stimulation fully converts M2 macrophages into M1 macrophages or, alternatively, merely induces the production of pro-inflammatory cytokines by M2 macrophages without a general conversion to an M1 phenotype, we analysed the effect of co-stimulation of M2 and M1 macrophages both functionally and phenotypically. c-IgG-TLR ligand co-stimulation induced similar amounts of TNF-α, IL-1β and IL-6 production by M2 and M1 macrophages (Supplementary Fig. 2c), indicating that after co-stimulation, M2 and M1 macrophages are indeed functionally very similar. In contrast, after co-stimulation, M2 macrophages were still phenotypically distinct from M1 macrophages, as indicated by elevated expression of four out of five tested M2 markers (that is, CD14, CD163, *SLC40A1* and *FOLR2*; Supplementary Fig. 3a,b). These data indicate that c-IgG-TLR ligand co-stimulation induces an inflammatory response by M2 macrophages, but does not induce a complete conversion to an M1 macrophage phenotype.

To verify the relevance of our findings with *in vitro*-generated macrophages, we tested the response of different types of primary M2 macrophages and alternative *in vitro* M2 models. Most importantly, we assessed the response of c-IgG-TLR ligand stimulation on CD14^+^CD163^+^ macrophages isolated from synovial fluid of RA patients with active disease (Supplementary Fig. 4). Indeed, c-IgG co-stimulation upregulated the production of pro-inflammatory cytokines by synovial fluid M2 macrophages from multiple patients, while IL-10 production was unaffected (Fig. 1d). Alternatively, we differentiated macrophages *in vitro* by incubating monocytes for 5 days in the presence of synovial fluid obtained from RA patients. Notably, cytokine responses of these monocyte-derived macrophages were very similar to M-CSF-differentiated M2 macrophages, with very little production of pro-inflammatory cytokines induced by individual c-IgG or TLR stimulation, but a strong upregulation after co-stimulation (Supplementary Fig. 5). In addition, c-IgG stimulation upregulated TLR-induced cytokine production by primary CD14^+^CD163^+^ macrophages isolated from the dermal layer of human skin (Supplementary Fig. 6), indicating that the observed effect is also functional in M2 macrophages that reside in non-rheumatic tissue. Finally, we tested the response of two alternative *in vitro* M2 models by differentiating M2 macrophages from monocytes using IL-10 or IL-4. IL-10-differentiated macrophages closely resembled M-CSF-differentiated M2 macrophages in phenotype (Supplementary Fig. 7a,b), as well as in their response to co-stimulation with TLR ligands and c-IgG (Supplementary Fig. 7c). IL-4-differentiated macrophages displayed a lower expression of M2 macrophage markers than M-CSF- and IL-10-differentiated macrophages (Supplementary Fig. 7a,b), which correlated with their restricted upregulation of pro-inflammatory cytokines (mainly TNF-α, but not IL-1β and IL-6) on co-stimulation with c-IgG (Supplementary Fig. 7c).

Taken together, these data demonstrate that in the presence of c-IgG, M2 macrophages show a strong pro-inflammatory response to TLR stimulation instead of an anti-inflammatory response, which is characterized by highly elevated production of TNF-α, IL-1β and IL-6.

### Response to co-stimulation is not altered in RA patients

**Figure 2:**
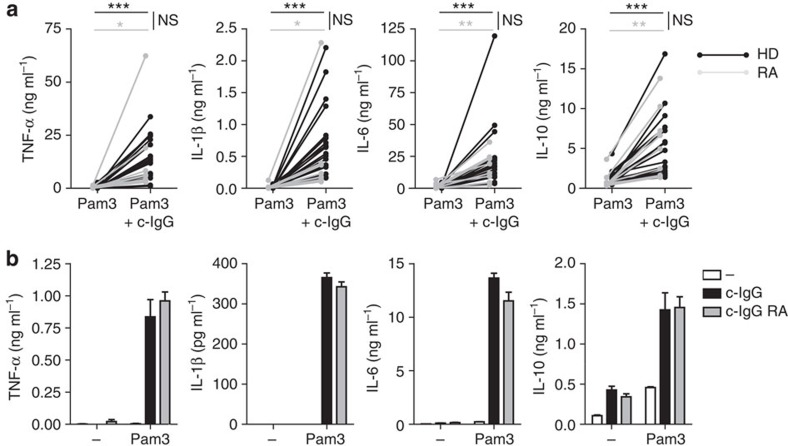
Synergistic upregulation of pro-inflammatory cytokines on c-IgG-TLR ligand co-stimulation does not differ between HD and RA patients. (**a**) M2 macrophages derived from monocytes of HD (*n*=20) or RA patients (*n*=9) were stimulated with Pam3CSK4 or Pam3CSK4 combined with c-IgG. Each pair of dots represents one donor. *P≪0.05, **P≪0.01, ***P≪0.001; NS, not significant, Kruskal Wallis test. (**b**) HD M2 macrophages were stimulated with Pam3CSK4 combined with HD c-IgG or c-IgG from RA patients. Data (mean+s.e.m.) are representative of four experiments with different donors. (**a**,**b**) After 24 h, cytokine levels were determined by ELISA. Experiments were performed in triplicate.

Previously, it has been shown that the response of myeloid cells from active RA patients can differ from those of healthy donors (HD)^25^, which may be caused by either intrinsic differences in RA patients or by the disease-induced inflammatory milieu. To determine whether the upregulation of pro-inflammatory cytokines differs between HD and RA patients, we compared cytokine production by M2 macrophages generated from monocytes of either HD or RA patients with active disease. Notably, no significant differences in c-IgG-induced upregulation of cytokines were observed between M2 macrophages of HD or active RA patients, either using Pam3CSK4 (Fig. 2a) or LPS (Supplementary Fig. 8a).

Besides differences in responses of myeloid cells, also the IgG molecules of RA patients and HD may differ in their glycosylation of the Fc part, which may lead to distinct antibody-mediated inflammatory responses^26^. To investigate the potential role of such IgG differences in co-stimulation of M2 macrophages, we compared cytokine production on (co-)stimulation with c-IgG from HD and with c-IgG from ACPA^+^ RA patients. No major differences in any of the measured cytokines were found between IgG from HD and ACPA^+^ RA patients, either by c-IgG stimulation alone or on co-stimulation with Pam3CSK4 (Fig. 2b) or LPS (Supplementary Fig. 8b). These data demonstrate that, despite potential differences in glycosylation, IgG from RA patients induces inflammatory responses by M2 macrophages in a similar manner as HD IgG. Taken together, these results indicate that M2 macrophages of RA patients respond similarly to c-IgG-TLR ligand co-stimulation as M2 macrophages of HD, and suggest that the mechanism responsible for c-IgG-TLR ligand-induced cytokine upregulation does not depend on cell-intrinsic or inflammation-associated differences.

### Enhanced cytokine transcription and caspase-1 activation

**Figure 3:**
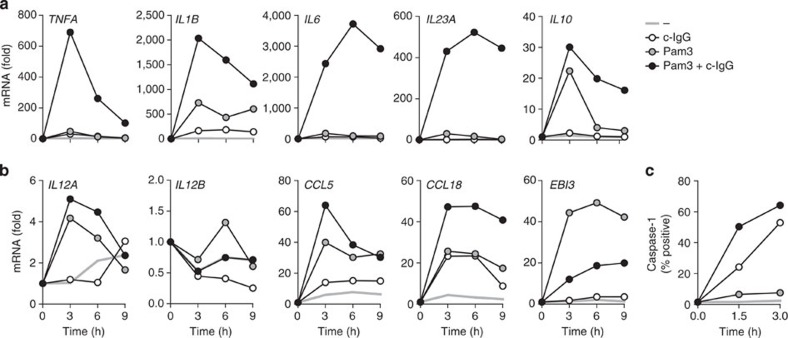
c-IgG-TLR ligand co-stimulation of M2 macrophages enhances pro-inflammatory cytokine transcription and caspase-1 activity. M2 macrophages were stimulated with Pam3CSK4, c-IgG or a combination and analysed for (**a**,**b**) mRNA expression of indicated genes (normalized to *GAPDH* expression, fold increase compared with unstimulated control) that was determined by quantitative RT–PCR or (**c**) caspase-1 activity, using caspase-1-binding compound FAM-YVAD-FMK at indicated time points. (**a**–**c**) Data are representative of three experiments with different donors.

Modulation of cytokine production can be orchestrated at different levels. To study whether the c-IgG-induced upregulation of cytokines is regulated at the transcriptional level, we analysed mRNA expression of the genes of interest. Indeed, c-IgG-Pam3CSK4 co-stimulation strongly and synergistically upregulated transcription of *TNFA*, *IL1B*, *IL6* and *IL23A*, while *IL10* transcription was only moderately increased (Fig. 3a). With the exception of IL-23, which was below protein detection limits, mRNA upregulation correlated with protein production (Fig. 1b), suggesting that the increased production of pro-inflammatory cytokines by M2 macrophages is regulated transcriptionally. Importantly, TLR-induced mRNA production of various other cytokines and chemokines, including *IL12A*, *IL12B*, *CCL5* and *CCL18*, was hardly affected by co-stimulation with c-IgG, while *EBI3* (encoding an IL-27/IL-35 subunit) was even downregulated (Fig. 3b). Importantly, these data demonstrate that c-IgG-TLR ligand co-stimulation of M2 macrophages does not result in general cytokine amplification, but instead specifically enhances production of particular pro-inflammatory cytokines.

In addition to human cells, we also determined whether c-IgG co-stimulation upregulated the transcription of pro-inflammatory cytokines in murine M2 macrophages, which we assessed by stimulating both IL-4- or IL-10-polarized bone marrow-derived macrophages with complexed murine IgG. In striking contrast to human M2 macrophages, c-IgG co-stimulation did not affect TLR2- or TLR4-induced transcription of *Tnf* by murine M2 macrophages (Supplementary Fig. 9). Co-stimulation with c-IgG did synergistically enhance the transcription of *Il1b* and *Il6*, but this effect was mainly observed on co-ligation with TLR2 and substantially less clear on co-ligation with TLR4 (Supplementary Fig. 9). These data suggest that c-IgG-induced cytokine production, especially TNF-α, is limited in murine compared with human M2 macrophages.

Remarkably, in human M2 macrophages, IL-1β on mRNA level was not upregulated to the same extent as on protein level (Figs 3a and 1b). This difference could be explained by regulation of caspase-1 activation, which is required for processing pro-IL-1β to active IL-1β^27^,^28^. Therefore, we examined caspase-1 activity in M2 macrophages on TLR ligand and/or c-IgG stimulation using caspase-1-binding compound FAM-YVAD-FMK. While stimulation with Pam3CSK4 alone had little effect, c-IgG stimulation strongly activated caspase-1, which was further amplified by Pam3CSK4 co-stimulation (Fig. 3c). Combined, these data indicate that c-IgG-TLR ligand co-stimulation upregulates the production of specific pro-inflammatory cytokines, by both upregulating transcription and by activating caspase-1.

### Co-stimulation of M2 macrophages promotes Th17 responses

**Figure 4:**
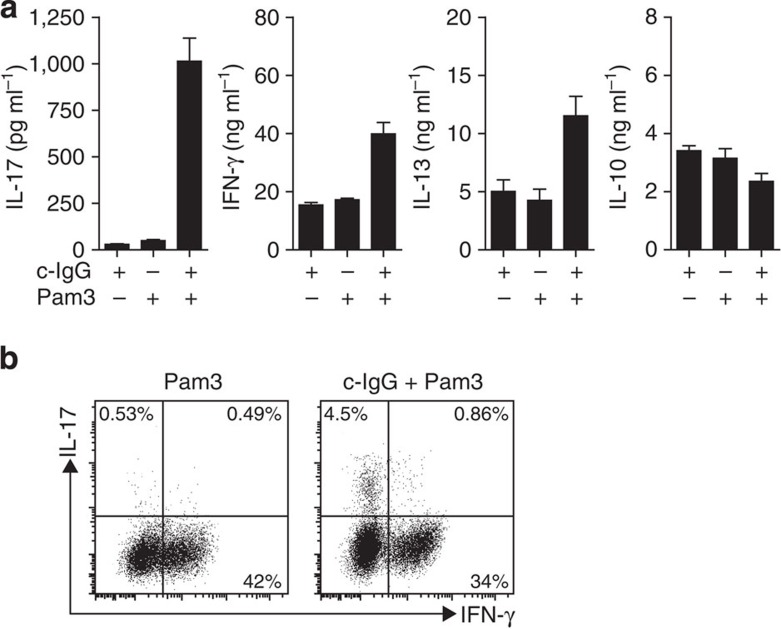
c-IgG-TLR ligand co-stimulation of M2 macrophages promotes Th17 responses. M2 macrophages were stimulated with Pam3CSK4, c-IgG or a combination and co-cultured with allogeneic CD4^+^ T cells. After T cell outgrowth, resting T cells were restimulated. (**a**) After 24 h, cytokine levels were determined by ELISA, mean+s.e.m. Experiments were performed in triplicate. (**b**) T cells were analysed for intracellular IL-17 and IFN-γ by flow cytometry. (**a**,**b**) Data are representative of three experiments with different donors.

Besides their crucial innate immune function that contributes to RA pathology via the production of pro-inflammatory cytokines, macrophages can also function as antigen-presenting cells that induce T helper (Th) cell polarization. RA is associated with a strong increase in activity of Th17 cells, both systemically and locally in inflamed synovial^5^,^6^,^7^,^29^. Human Th17 polarization is thought to be dependent on IL-1β, IL-6 and TNF-α^30^,^31^, which were all strongly induced by M2 macrophages after c-IgG-TLR ligand co-stimulation. To determine whether co-stimulation of M2 macrophages indeed affects Th polarization, we co-cultured stimulated macrophages with CD4^+^ T cells and quantitatively determined the secretion of hallmark cytokines IL-17 (Th17), IFN-γ (Th1), IL-13 (Th2) and IL-10. Strikingly, c-IgG-Pam3CSK4 co-stimulation strongly increased IL-17 production (Fig. 4a). In contrast, IFN-γ, IL-13 and IL-10 production was hardly affected by co-stimulation with c-IgG (Fig. 4a). We also determined the production of IL-17 and IFN-γ by intracellular cytokine staining, which confirmed the enhanced Th17 activation in response to c-IgG-Pam3CSK4 co-stimulation (Fig. 4b). Collectively, these experiments demonstrate that c-IgG-TLR ligand co-stimulation selectively promotes Th17 responses induced by human M2 macrophages. Since M2 macrophages in general are associated with Th2 and regulatory T cell responses^2^, these data suggest a major alteration in polarization of M2 macrophage-induced adaptive immunity.

### Syk inhibition blocks FcγR-induced cytokine production

**Figure 5:**
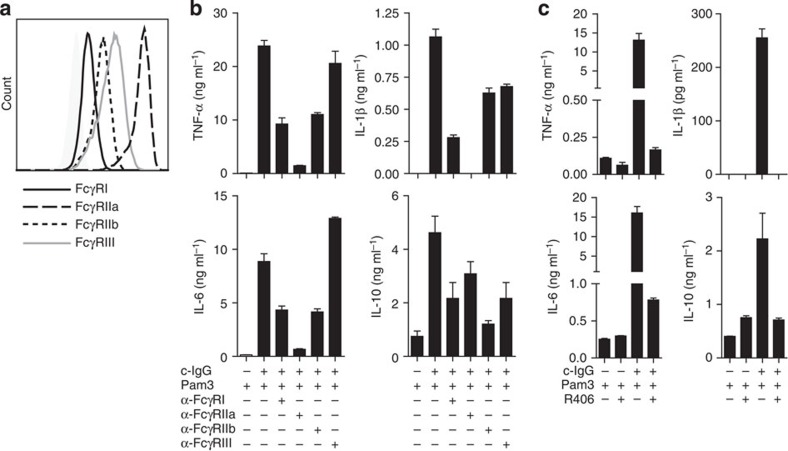
Synergistic upregulation of pro-inflammatory cytokines on c-IgG-TLR ligand co-stimulation is dependent on FcγRs and Syk. (**a**) FcγR expression (10log fluorescence intensity) on unstimulated M2 macrophages was analysed by flow cytometry. (**b**) Before stimulation, M2 macrophages were incubated with blocking antibodies against indicated FcγRs or (**c**) 1 μM of Syk inhibitor R406. (**a**–**c**) Data are representative of at least three experiments with different donors. (**b**,**c**) After 24 h, cytokine levels were determined by ELISA, mean+s.e.m. Experiments were performed in triplicate.

The main receptors for IgG on macrophages belong to the family of Fc gamma receptors (FcγRs)^32^. As shown in Fig. 5a, M2 macrophages expressed FcγRI, IIa, IIb and III. To determine whether FcγRs are responsible for the synergistic cytokine upregulation after c-IgG-TLR ligand co-stimulation, we blocked different FcγRs with specific antibodies during M2 stimulation and analysed cytokine production. Blocking of FcγRIIa almost completely inhibited c-IgG-induced upregulation of TNF-α, IL-1β and IL-6 (Fig. 5b). In addition, upregulation of these cytokines was partially inhibited by anti-FcγRI and anti-FcγRIIb (Fig. 5b). In contrast to the pro-inflammatory cytokines, upregulation of IL-10 was mainly blocked by anti-FcγRIIb (Fig. 5b), which corresponds to the anti-inflammatory role of this receptor^32^. Combined, these data indicate that c-IgG-TLR ligand-induced cytokine upregulation by M2 macrophages depends on cross-talk of TLRs with the family of FcγRs, with a main role for FcγRIIa for the induction of pro-inflammatory cytokines.

FcγR-TLR cross-talk on M2 macrophages specifically upregulated TNF-α, IL-1β and IL-6, which are all known to be involved in RA pathology^5^,^6^,^7^. Hence, inhibition of FcγR function on M2 macrophages could be of potential therapeutic interest, since this would specifically block production of pro-inflammatory cytokines while leaving TLR-induced IL-10 production intact (see also Fig. 1a). Many of the effects induced by activating FcγRs, including FcγRI and IIa, depend on signalling via spleen tyrosine kinase (Syk)^32^. To determine whether Syk is also required for FcγR-TLR cross-talk in M2 macrophages, we blocked Syk using R406 (also known as tamatinib), a kinase inhibitor that is currently also being tested for therapeutical use in RA patients^33^. Strikingly, in our experiments, R406 almost completely blocked c-IgG-TLR ligand-induced upregulation of TNF-α, IL-1β and IL-6 (Fig. 5c). Importantly, inhibition by R406 was highly specific, since it selectively blocked synergistic cytokine production on FcγR-TLR co-stimulation, but did not substantially affect cytokine production induced by TLR stimulation alone (Fig. 5c). Thus, R406 seemed to restore the anti-inflammatory profile of M2 macrophages, characterized by production of mainly IL-10 but very little pro-inflammatory cytokines. These data not only demonstrate that production of pro-inflammatory cytokines induced by FcγR-TLR cross-talk is Syk dependent, but also indicate that FcγR-induced inflammation by M2 macrophages can be counteracted using therapeutic kinase inhibitors.

## Discussion

M2 macrophages are generally considered to attenuate inflammation in tissues through the induction of anti-inflammatory responses. In contrast, in this study, we found that exposure of M2 macrophages to the particular combination of c-IgG and TLR ligands, as occurs in the synovia of the majority of RA patients, strongly promotes inflammation. This pro-inflammatory response is characterized by selective amplification of TNF-α, IL-1β, IL-6 and Th17 polarization, which are all strongly implicated in RA pathology. The main receptor for the c-IgG-induced effects is the low-affinity IgG receptor FcγRIIa, which amplifies pro-inflammatory cytokine production in a Syk-dependent manner by both enhancing *TNFA*, *IL1B*, *IL6* and *IL23A* transcription as well as by activating caspase-1, leading to increased production of functional IL-1β. Together, these results suggest that in the context of RA, human M2 macrophages may promote instead of inhibiting inflammation.

Under most conditions, M2 macrophages have been found to predominantly express anti-inflammatory cytokines such as IL-10 and TGF-β^2^,^3^,^34^. In contrast, previous immunohistochemistry data already showed that in synovia of RA patients, over 80% of CD163^+^ macrophages stain positive for the pro-inflammatory cytokine TNF-α^35^, which corroborates our *ex vivo* and *in vitro* findings that in RA, M2 macrophages promote instead of attenuating inflammation. Notably, we did not observe differences in FcγR-TLR-induced cytokine production between M2 macrophages generated from active RA patients and HD. This strengthens the idea that FcγR-TLR cross-talk is a general mechanism of the human immune system to promote inflammation, which is further supported by our finding that this mechanism is also functional in M2 macrophages that reside in non-rheumatic tissue such as the dermal layer of the skin. The physiological function of this ‘inflammatory switch’ of M2 macrophages on exposure to IgG immune complexes is still speculative, but FcγR-TLR cross-talk may be advantageous to counteract bacterial infections. Bacteria are rapidly IgG opsonized during infection, leading to complex formation that can activate FcγRs, which acts as an additional signal to efficiently combat extracellular pathogens^36^,^37^. In RA synovia, this signal may be induced undesirably, as a result of the presence of IgG autoantibodies that form immune complexes after recognition of self-antigens. As a consequence, the conventional function of M2 macrophages, that is, preventing disproportionate immune activation and mediating tissue repair, is abrogated, which may thereby underlie excessive inflammation as observed in RA patients. In addition to RA, the pro-inflammatory response of M2 macrophages may occur in other diseases that involve both IgG immune complexes and macrophages, such as systemic sclerosis^38^ and systemic lupus erythematosus^39^. As such, our data support the concept that, instead of merely being a consequence of immune activation, IgG autoantibodies actively contribute to pathology of these autoimmune diseases^22^.

Notably, FcγR-TLR cross-talk-dependent cytokine induction appears to be restricted in murine compared with human M2 macrophages. Most importantly, in mice, FcγR-TLR cross-talk did not affect the production of TNF-α, which is a pivotal cytokine in RA pathogenesis and currently the most successful target for the therapy of RA patients^5^. Considering these differences, FcγR-TLR cross-talk seems more important in the pathogenesis of human disease and most likely plays a subordinate role in mouse models for arthritis. Mechanistically, this difference between human and mouse may be explained by the difference in expression of FcγRIIa, which was the main receptor responsible for c-IgG-induced production of pro-inflammatory cytokines by human M2 macrophages, but is not expressed in mice^32^.

The pro-inflammatory response of M2 macrophages induced by FcγR-TLR cross-talk could be completely converted to an anti-inflammatory response using Syk inhibitor R406. Importantly, R406-prodrug fostamatinib disodium (R788) shows clinical efficacy in RA patients^33^. However, the mechanism through which it inhibits inflammation in RA patients is still not completely clear^33^. Our data may provide a possible mechanistic explanation for the therapeutic efficacy of Syk inhibition in RA patients, that is, by restoring the anti-inflammatory and subsequent tissue-repairing function of M2 macrophages. In addition, Syk inhibition may interfere with induction of inflammation by other cell types, since FcγR-TLR cross-talk also (moderately) enhanced the production of pro-inflammatory cytokines by M1 macrophages.

Taken together, our results indicate that, while M2 macrophages induce anti-inflammatory responses in diseases that do not involve immune complexes, we may need to re-evaluate their role in diseases such as RA, where instead of counteracting inflammation, M2 macrophages may actually contribute to pathology. From a therapeutic point of view, targeting of FcγR-TLR cross-talk to restore the anti-inflammatory function of M2 macrophages may be a valuable tool to attenuate inflammation in IgG immune complex-associated diseases such as RA, systemic sclerosis and systemic lupus erythematosus.

## Methods

### *In vitro* macrophage differentiation

Monocytes were isolated from heparinized peripheral blood from HD or nine RA patients (active disease according to the American College of Rheumatology criteria, ACPA^+^ and/or rheumatoid factor^+^, non-biologicals treated) by density gradient centrifugation on Lymphoprep (Nycomed) and Percoll (Pharmacia). All patients gave written informed consent to participate in the study as approved by the Medical Ethics Committee of the Academic Medical Center, Amsterdam.

Macrophages were generated by culturing monocytes for 6 days in IMDM (Gibco) containing 5% fetal bovine serum (FBS; Invitrogen) and 86 μg ml^−1^ gentamicin (Duchefa), supplemented with 50 ng ml^−1^ recombinant human M-CSF (Immunotools) for M2 macrophages or 500 U ml^−1^ recombinant human GM-CSF (Schering-Plough) for M1 macrophages. At day 2 or 3, half of the medium was replaced by new medium containing cytokines. For M0 macrophages, monocytes were cultured for 6 days in the presence of 5% pooled human serum, without any FBS or cytokines. At day 2 or 3, half of the medium was replaced by new medium containing human serum. To mimic macrophage differentiation from monocytes in the synovial microenvironment, monocytes were cultured for 5 days in the presence of 10% pooled synovial fluid from 20 RA patients with active disease (according to the American College of Rheumatology criteria), as described previously^40^. For M2_IL-10_ or M2_IL-4_, monocytes were cultured for 6 days in the presence of 50 ng ml^−1^ recombinant human IL-10 or 10 U ml^−1^ recombinant human IL-4 (both from Miltenyi Biotec), respectively. At day 2 or 3, half of the medium was replaced by new medium containing cytokines.

### Primary RA macrophages

Synovial fluid was collected from inflamed joints of 6 RA patients (active disease according to the American College of Rheumatology criteria, non-biologicals treated). From these samples, cells were collected by centrifugation and either stored in liquid nitrogen or used directly. Cells were stained using anti-CD3-FITC (SK7; 1:50; BD Biosciences), anti-CD19-FITC (HD37; 1:50; Dako), anti-CD20-FITC (L27; 1:50; BD Biosciences), anti-CD56-FITC (NCAM16.2; 1:50; BD Biosciences), anti-CD66b-FITC (CLB-B13.9; 1:50; Sanquin), anti-CD11c-PECy7 (3.9; 1:200; eBioscience), anti-CD14-phycoerythrin (PE; MϕP9; 1:100; BD Biosciences) and anti-CD163-allophycocyanin (GHI/61; 1:150; BioLegend) and FACS (fluorescence-activated cell sorting) sorted (FACSAria, BD Biosciences). CD3^−^CD19^−^CD20^−^CD56^−^CD66b^−^CD11c^+^CD14^+^CD163^+^ cells were stimulated as indicated.

### Primary dermal macrophages

For isolation of primary dermal macrophages, human skin specimens were obtained from healthy subjects undergoing breast or abdominal reduction surgery. Dermis and epidermis were enzymatically separated by overnight incubation at 4 °C with 2 mg ml^−1^ dispase II (Roche). Dermis was then treated with 5 mg ml^−1^ collagenase D and 7 μg ml^−1^ DNase (both from Roche) for 1.5 h at 37 °C. After single-cell separation using 70 μm cell strainers (Greiner Bio-One), cells were stained using anti-HLA-DR-PerCP (G46-6; 1:5; BD Biosciences), anti-CD11c-PECy7, anti-CD14-FITC (MϕP9; 1:100; BD Biosciences) and anti-CD163-allophycocyanin and FACS sorted (FACSAria, BD Biosciences). HLA-DR^+^CD11c^+^CD14^+^CD163^−^ and HLA-DR^+^CD11c^+^CD14^+^CD163^+^ cells were stimulated as indicated or lysed directly for mRNA expression analysis.

### Flow cytometry

For phenotyping, macrophages were stained with anti-CD14-PE and anti-CD163-allophycocyanin in phosphate-buffered saline (PBS) containing 1% bovine serum albumin (BSA), 2% FBS, 1% pooled human serum (Lonza) and 0.1% sodium azide. For CD14 and CD163 expression analysis after co-stimulation, cells were harvested using TrypLE Select (Invitrogen) and stimulated in 48-well plates (Greiner Bio-one) for 24 h in IMDM without phenol red (Gibco). Fluorescence of the cells was assessed by flow cytometry (Canto II, BD Biosicences).

### Stimulation

Macrophages were harvested at day 6 by gentle scraping and stimulated (30,000–50,000 cells per well) with 10 μg ml^−1^ Pam3CSK4 (Invivogen), 100 ng ml^−1^ LPS (from *Escherichia coli* 0111:B4; Sigma-Aldrich) or 100,000 dead synovial fibroblasts combined with c-IgG. Synovial fibroblasts were derived from knee biopsies of RA patients and expanded *in vitro* in DMEM (Invitrogen) supplemented with 10% FBS. Fibroblasts were stored in liquid nitrogen. To obtain dead cells for stimulation, cells were thawed and subsequently damaged and killed by three cycles of freeze–thawing. For c-IgG stimulation, 96-well high-affinity Maxisorp plates (Nunc) were coated with 2 μg ml^−1^ IgG from HD (Nanogam; Sanquin Blood Supply) or RA patients (see below) and diluted in PBS for 1 h at room temperature, followed by blocking with PBS containing 10% FBS for 1 h at 37 °C. IgG from five ACPA^+^ RA patients was isolated from plasma, using protein G antibody affinity chromatography (GE Healthcare Life Sciences), and pooled. After stimulation, supernatants were harvested after 24 h and stored at −20 °C until analysis by enzyme-linked immunosorbent assay (ELISA). For comparison between primary RA macrophages from different patients, or between HD and RA patients from different experiments, values for cytokine levels were normalized to 50,000 cells per well.

### Th polarization

Memory CD4^+^ T cells were isolated using MACS isolation (Miltenyi Biotec) with anti-CD45RO-PE (Dako) and anti-PE beads (Miltenyi Biotec). For *in vitro* differentiation of T cells, 50,000 M2 macrophages were stimulated with Pam3CSK4 and/or c-IgG and co-cultured with 50,000 allogeneic memory CD4^+^ T cells, in the presence of 10 pg ml^−1^ *Staphylococcus aureus* enterotoxin B (SEB; Sigma-Aldrich). After about 4 days, cells were transferred to 96-well flat-bottom culture plates (Greiner Bio-One). Every 2 days, half of the medium was replaced by IMDM (Lonza) containing 10% FBS and 20 U ml^−1^ recombinant human IL-2 (Chiron) and wells were split if necessary. For cytokine analysis, resting T cells (by about day 12) were restimulated (100,000 per well) with 1 μg ml^−1^ anti-CD3 (1XE; 1:2,000; Sanquin Blood Supply) and 1 μg ml^−1^ anti-CD28 (15E8; 1:2,000; Sanquin Blood Supply). Supernatants were harvested after 24 h and stored at −20 °C until analysis by ELISA. For intracellular cytokine staining, resting T cells were restimulated with 100 ng ml^−1^ phorbol myristate acetate, 1 μg ml^−1^ ionomycin and 10 μg ml^−1^ brefeldin A (all Sigma-Aldrich) for 6 h. Cells were washed, fixated with 4% formaldehyde (Sigma-Aldrich) for 15 min, washed again, permeabilized with 0.5% saponin (Calbiochem) in PBS containing 0.5% BSA (PAA) and 0.1% sodium azide (Merck). Cells were incubated with anti-IL-17-biotin (eBio64DEC17; 1:50; eBioscience) followed by streptavidin-PE (1:200; BD Pharmingen) and anti-IFNγ-FITC (25723.11; 1:10; BD Biosciences) for 30 min at room temperature and analysed by flow cytometry.

### ELISA

Cytokine levels in supernatants were measured by ELISA, using antibody pairs for the following cytokines: IL-1β (CT213-c; 1:400 and CD213-d; 1:400), IL-6 (CD205-c; 1:200 and CD205-d; 1:200), IL-13 (QS-13; 1:200 and LM-1; 1:100), IL-23 (CD517-c; 1:400 and C8.6; 1:4,000), IFN-γ (MD2; 1:500 and MD1; 1:500; all from U-CyTech), IL-10 (JES3-9D7; 1:1,000 and JES3-12G8; 1:1,000; BD Pharmingen), TNF-α (MAb1; 1:500 and MAb11; 1:250) and IL-17A (eBio64cap17; 1:1,000 and eBio64dec17; 1:1,000; both from eBioscience).

### Quantitative RT–PCR

For mRNA-level analysis, cells were lysed at the indicated time points, after which mRNA extraction was performed using NucleoSpin RNA Isolation Kit (Macherey-Nagel) and cDNA synthesis using First Strand cDNA Synthesis Kit (Fermentas). Quantitative RT–PCR (iCycler iQ Multi-Color Real Time PCR Detection System; Bio-Rad), was performed using SYBR green (Bio-Rad) and primer pairs as listed in Supplementary Table 1. mRNA levels were normalized to housekeeping gene expression (2^*C*t(*housekeeping*)−*C*t(target)^), and folds were calculated compared with an unstimulated control sample (*t*=0 h).

### FLICA

Caspase-1 activation was determined using caspase-1-binding compound FAM-YVAD-FMK from FLICA Apoptosis Detection Kit for Caspase-1 (Immunochemistry Technologies) according to the manufacturer’s guidelines. Fluorescence of the cells was assessed by flow cytometry.

### FcγR blockade and Syk inhibition

FcγR expression was determined by staining M2 macrophages with 5 μg ml^−1^ anti-FcγRI (CD64; 10.1; BD Biosciences), anti-FcγRIIa (CD32a; IV.3; StemCell Technologies), anti-FcγRIIb (CD32b; 2B6; MacroGenics) or anti-FcγRIII (CD16; 3G8; BD Biosciences) followed by PE-conjugated goat anti-mouse antibody (1:200; Jackson ImmunoResearch), in PBS containing 0.5% BSA and 0.1% sodium azide. Cells were analysed by flow cytometry. FcγRs were blocked by incubating M2 macrophages with 20 μg ml^−1^ anti-FcγRI/IIa/IIb/III for 30 min at 37 °C, after which stimuli and culture medium were added resulting in a final antibody concentration of 5 μg ml^−1^. Syk was inhibited by incubating M2 macrophages with 1 μM R406 (Selleckchem), or the corresponding volume of dimethylsulphoxide (DMSO; Sigma-Aldrich), for 2 h at 37 °C before stimulation.

### Mouse bone marrow-derived macrophages

Bone marrow was collected from 12- to 16-week-old female wild-type C57BL/6 mice (experiments approved by the animal experiments committee of the Academic Medical Center, Amsterdam) and cultured in 100 mm cell culture dishes (BD Falcon) in IMDM (Lonza) supplemented with 10% FBS, 86 μg ml^−1^ gentamicin and 40 ng ml^−1^ recombinant mouse M-CSF (Biolegend). At day 3, fresh medium containing M-CSF was added. At day 6, M-CSF and either 10 ng ml^−1^ recombinant mouse IL-10 or 20 ng ml^−1^ recombinant mouse IL-4 (both from eBioscience) were added. At day 7, cells were harvested and stimulated as described for human macrophages, using 2 μg ml^−1^ mouse IgG (Equitech-Bio) coated in Maxisorp plates. Cells were analysed by quantitative RT–PCR as described; primer pairs are listed in Supplementary Table 2.

### Data analysis

Data were analysed for statistical significance using Wilcoxon matched-pair test or Kruskal Wallis test (followed by Dunn’s multiple comparison test) with GraphPad Prism version 5.01 software (GraphPad Software).

## Author contributions

L.T.C.V. and J.d.D. devised the concept. L.T.C.V., D.L.P.B., M.L.K., E.C.d.J. and J.d.D. designed the research and analysed the data. L.T.C.V., I.S.H., T.R., F.J.M.M. and T.M.M.v.C. performed the research. T.R., M.C.T. and J.B.V. contributed reagents or materials. L.T.C.V., E.C.d.J. and J.d.D. wrote the paper.

## Additional information

**How to cite this article**: Vogelpoel, L. T. C. *et al.* Fc gamma receptor-TLR cross-talk elicits pro-inflammatory cytokine production by human M2 macrophages. *Nat. Commun.* 5:5444 doi: 10.1038/ncomms6444 (2014).

## Supplementary Material

Supplementary Information Supplementary Figures 1-9, Supplementary Tables 1-2.
