# Supplementary material for: Fc gamma receptor-TLR cross-talk elicits pro-inflammatory cytokine production by human M2 macrophages
Source: Nat Commun. 2014 Nov 13;5:5444. doi: 10.1038/ncomms6444 (PMC4243215; doi:10.1038/ncomms6444)
Supplement: Supplementary Information — Supplementary Figures 1-9, Supplementary Tables 1-2. [file ncomms6444-s1.pdf]

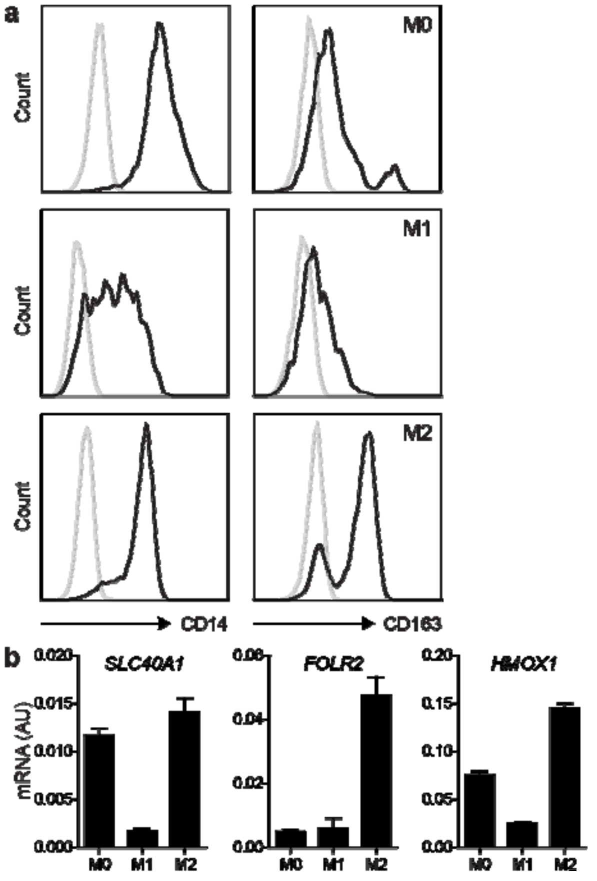

Supplementary Figure 1. Macrophage phenotype. (**a**) Expression of CD14 and CD163 on unstimulated M0 (control macrophages), M1, or M2 macrophages was analyzed by flow cytometry. Grey lines represent the corresponding background of unstained cells. (**b**) mRNA expression of unstimulated M0, M1, or M2 macrophages was analyzed for *SLC40A1*, *FOLR2*, and *HMOX1* (M2 markers), determined by quantitative RT-PCR. Expression was normalized to housekeeping genes (geometric mean of *GAPDH*, *B2M*, and *UBC* expression).

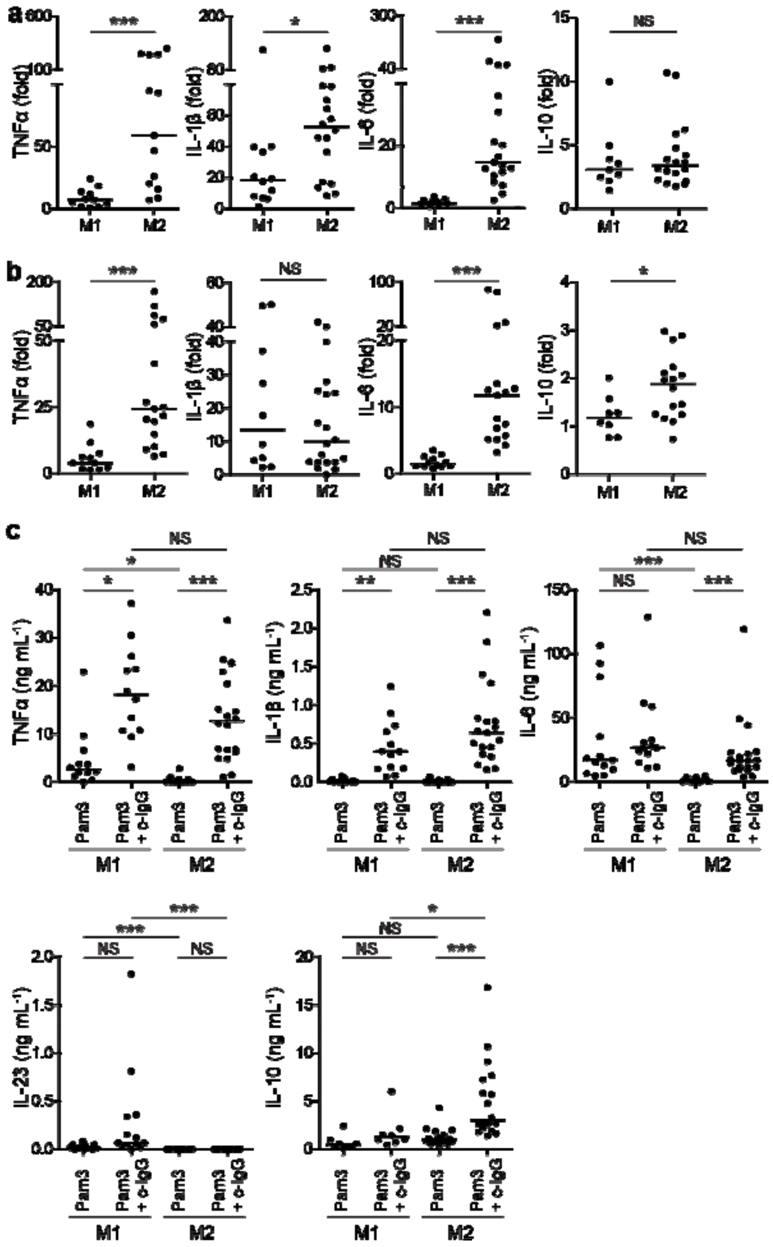

Supplementary Figure 2. Relative cytokine response. Folds of cytokine production (determined by ELISA) of M1 or M2 macrophages stimulated with Pam3CSK4 (**a**) or LPS (**b**) combined with c-IgG, versus Pam3CSK4 or LPS only. Each dot represents one donor, line represents median. *P < 0.05, ***P < 0.001, NS not significant, Mann Whitney U test. (**c**) M1 and M2 macrophages were stimulated with Pam3CSK4 or Pam3CSK4 combined with c-IgG. Each dot represents one donor, line represents median. *P < 0.05, **P < 0.01, ***P < 0.001, NS not significant, Kruskal Wallis test (followed by Dunn's multiple comparison test) .

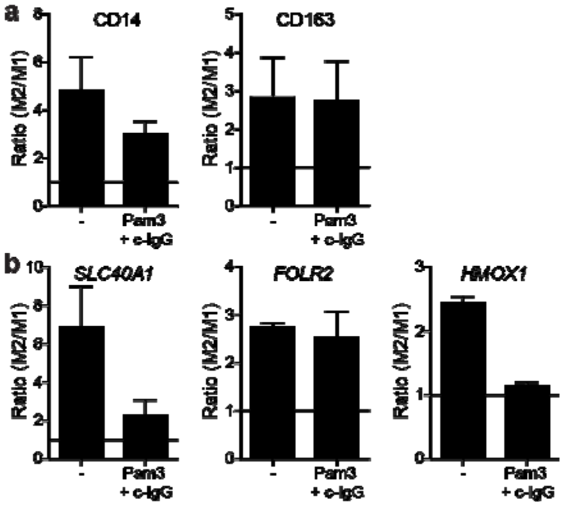

Supplementary Figure 3. Macrophage phenotype after c-IgG-TLR ligand co-stimulation. M1 and M2 macrophages were stimulated with Pam3CSK4 combined with c-IgG. After 24h cells were analyzed for CD14 and CD163 protein expression, determined by flow cytometry (**a**) and *SLC40A1*, *FOLR2*, and *HMOX1* mRNA expression, determined by quantitative RT-PCR (**b**). Data are presented as the ratio between expression of M2 over M1 macrophages (for protein expression: after background subtraction). Values above one (indicated by line) indicate elevated expression in M2 macrophages.

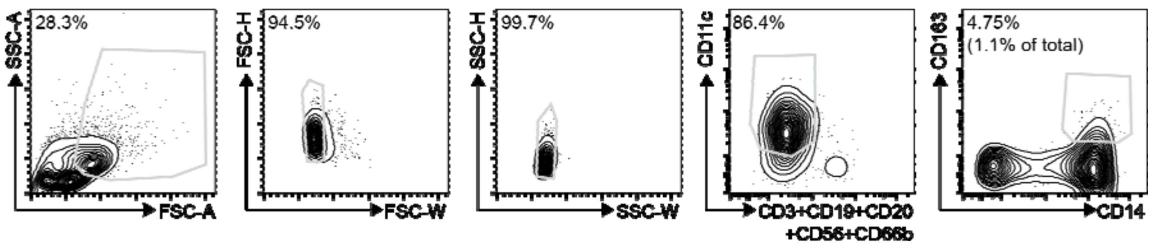

Supplementary Figure 4. Primary RA macrophages. Gating strategy of sorting of $CD163^+$ cells from RA synovial fluid. After single cell selection, $CD3^-CD19^-CD20^-CD56^-CD66b^-CD11c^+CD14^+CD163^+$ cells were sorted and used for analysis. One representative example.

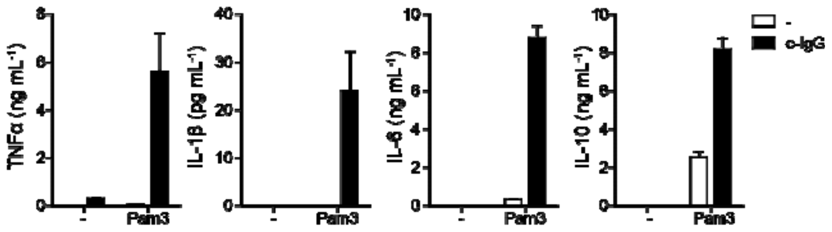

Supplementary Figure 5. RA synovial fluid-differentiated macrophages. Monocytes were differentiated in the presence of 10% synovial fluid pooled from 20 RA patients. Cells were stimulated with Pam3CSK4, c-IgG or a combination. After 24h cytokine levels were determined by ELISA, mean+s.e.m. Data are representative of four experiments, performed in triplicate, with different donors.

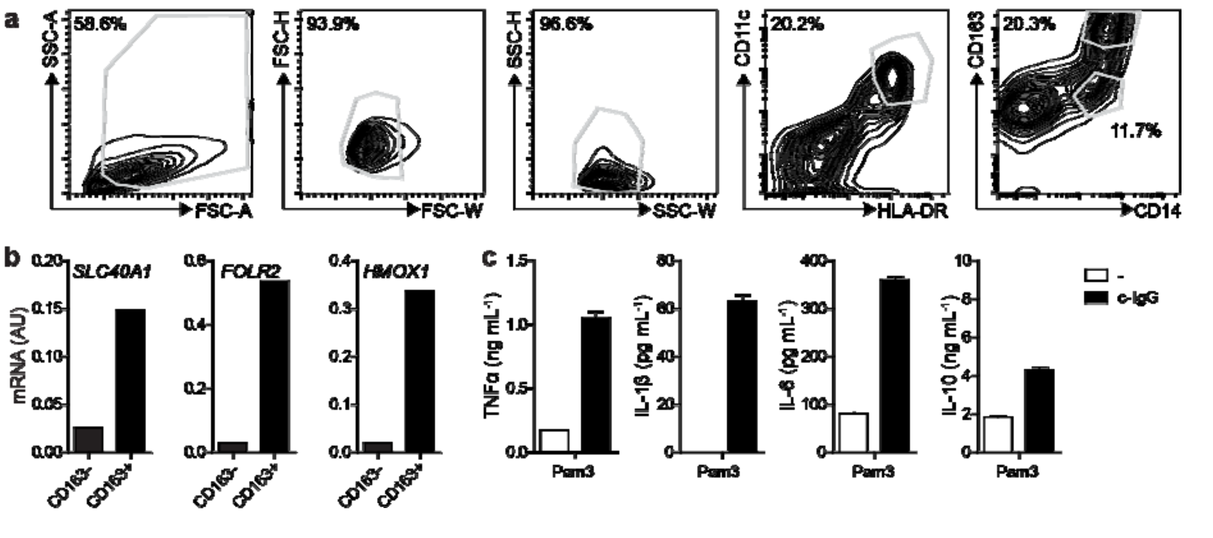

Supplementary Figure 6. Primary dermal macrophages. (**a**) Gating strategy of sorting of CD163$^+$ cells from dermis. After single cell selection, HLA-DR$^+$CD11c$^+$CD14$^+$CD163$^+$ cells were sorted and used for analysis. One representative example. (**b**) mRNA expression of unstimulated CD163$^+$ and CD163$^-$ cells was analyzed for *SLC40A1*, *FOLR2*, *HMOX1* (M2 markers), determined by quantitative RT-PCR. Expression was normalized to housekeeping genes (geometric mean of *GAPDH*, *B2M,* and *UBC* expression). (**c**) CD163$^+$ cells were stimulated with Pam3CSK4 or Pam3CSK4 combined with c-IgG. After 24h cytokine levels were determined by ELISA, mean+s.e.m. Data are representative of two experiments, performed in triplicate, with different donors.

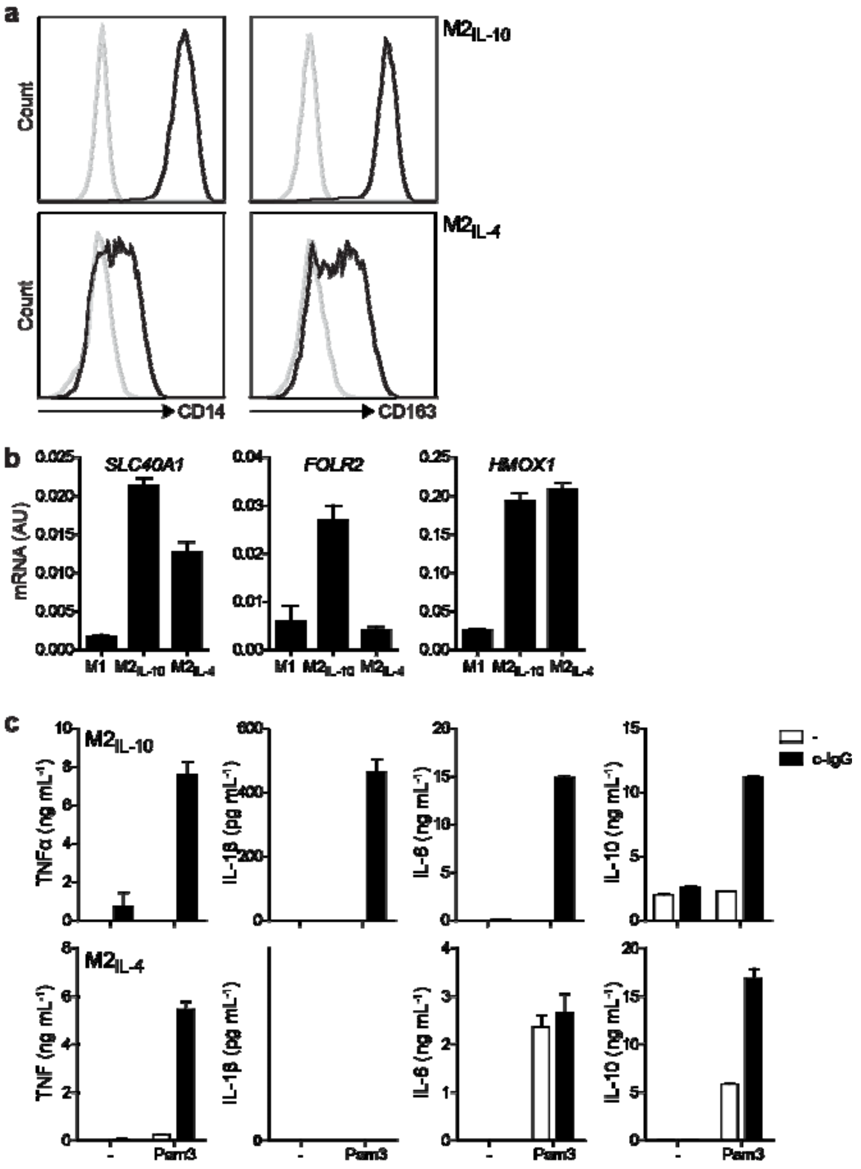

Supplementary Figure 7. M2$_{IL-10}$ and M2$_{IL-4}$ macrophages. (**a**) Expression of CD14 and CD163 on unstimulated IL-10-differentiated M2 macrophages (M2$_{IL-10}$) or IL-4-differentiated M2 macrophages (M2$_{IL-4}$) was analyzed by flow cytometry. Grey lines represent the corresponding background of unstained cells. (**b**) mRNA expression of unstimulated M1, M2$_{IL-10}$, and M2$_{IL-4}$ macrophages was analyzed for *SLC40A1*, *FOLR2*, and *HMOX1* (M2 markers), determined by quantitative RT-PCR. Expression was normalized to housekeeping genes (geometric mean of *GAPDH*, *B2M*, and *UBC* expression). (**c**) M2$_{IL-10}$ (top panel) and M2$_{IL-4}$ (bottom panel) macrophages were stimulated with Pam3CSK4, c-IgG or a combination. After 24h cytokine levels were determined by ELISA, mean+s.e.m. Data are representative of three experiments, performed in triplicate, with different donors.

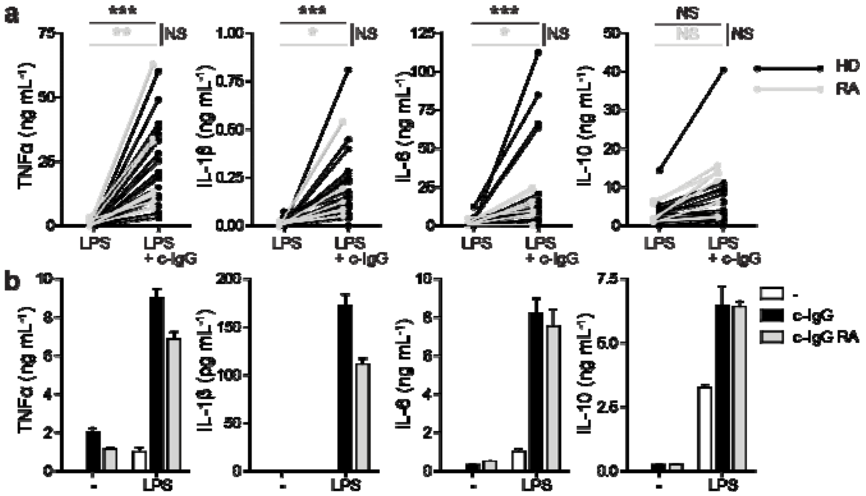

Supplementary Figure 8. Co-stimulation of M2 macrophages from RA patients. (**a**) M2 macrophages derived from monocytes of HD (*n*=20) or RA patients (*n*=9) were stimulated with LPS or LPS combined with c-IgG. Each pair of dots represents one donor. **P* < 0.05, ***P* < 0.01, ****P* < 0.001, NS not significant, Kruskal Wallis test. (**b**) HD M2 macrophages were stimulated with LPS combined with HD c-IgG or c-IgG from RA patients. Data (mean+s.e.m.) are representative of four experiments with different donors. (**a** and **b**) After 24h cytokine levels were determined by ELISA. Experiments were performed in triplicate.

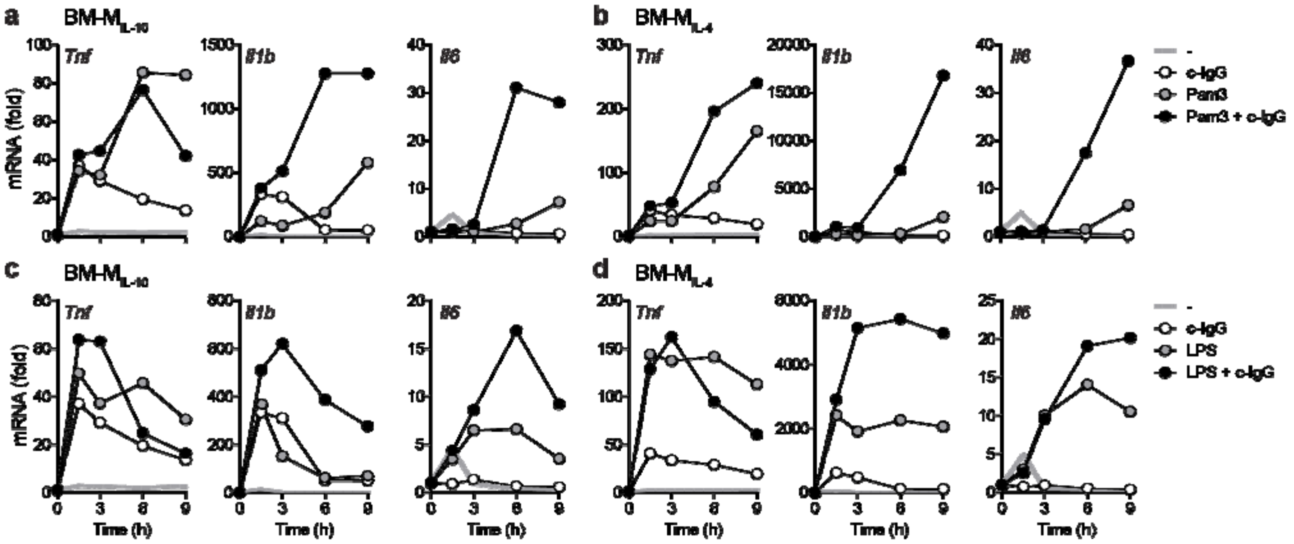

Supplementary Figure 9. Co-stimulation of bone marrow-derived macrophages. Murine bone marrow was differentiated into macrophages and polarized using IL-10 ($BM\text{-}M_{IL\text{-}10}$; **a** and **c**) or IL-4 ($BM\text{-}M_{IL\text{-}4}$; **b** and **d**). Cells were stimulated using Pam3CSK4 (**a** and **b**) or LPS (**c** and **d**), c-IgG or a combination. Cells were lysed at indicated time points and analyzed for mRNA expression of indicated genes, determined by quantitative RT-PCR (normalized to geometric mean of *Actb*, *Gapdh*, and *Hprt* expression; fold increase compared to unstimulated control).

Supplementary Table 1. Primers for quantitative RT-PCR (human genes)

Target mRNA	Forward primer (5'-3')	Reverse primer (5'-3')
B2M	AAGATTCAGGTTTACTCACGTC	TGATGCTGCTTACATGTCTCG
CCL5	AACCCAGCAGTCGTCTTTGT	AGTTGATGTACTCCCGAACCC
CCL18	CTTGTCCTCGTCTGCACCAT	CCCTCAGGCATTCAGCTTCA
EBI3	CGTGCCTTTCATAACAGAGCA	GACGTAGTACCTGGCTCGG
FOLR2	CCTGCAGGGACAGAAAGACA	CCAGGGACTGCATTGGTCAT
GAPDH	GAAGGTGAAGGTCGGAGTC	GAAGATGGTGATGGGATTT
HMOX1	CTGCGTTCCTGCTCAACATC	ATCTTGCACTTTGTTGCTGGC
IL1B	TTTGAGTCTGCCCAGTTCCC	TCAGTTATATCCTGGCCGCC
IL6	TGACAAACAAATTCGGTACATCCT	AGTGCCTCTTTGCTGCTTTCAC
IL10	ATGCTTCGAGATCTCCGAGA	AAATCGATGACAGCGCCGTA
IL12A	AGTGCCGGCTCAGCATGTGT	GTGGCCACGGGGAGGTTTCT
IL12B	ACGTTTCACCTGCTGGTGGCT	CTCCGCACGTCACCCCTTGG
IL23A	GTGGGACACATGGATCTAAGAGAAG	TTTGCAAGCAGAACTGACTGTTG
SLC40A1	TATTCATGCCTGGAAGCCCC	TTCTAGCAGCAATGACGCCT
TNFA	GGCTCCAGGCGGTGCTTG	CAGATAGATGGGCTCATACCA
UBC	GTACCCTGTCTGACTACAACAT	GTGATGGTCTTGCCAGTGAG

Supplementary Table 2. Primers for quantitative RT-PCR (mouse genes)

Target mRNA	Forward primer (5’-3’)	Reverse primer (5’-3’)
Actb	GTGGGCCGCTCTAGGCACCAA	CTCTTTGATGTCACGCACGATTTC
Gapdh	GTGCTGAGTATGTCGTGGAGTCTAC	TTGCTGACAATCTTGAGTGAGTTG
Hprt	TCCTCCTCAGACCGCTTTT	CATAACCTGGTTCATCATCGC
Il1b	GCCTCGTGCTGTCGGACCCAT	GCAGGGTGGGTGTGCCGTC
Il6	GGTGACAACCACGGCCTTCCC	TTAAGCCTCCGACTTGTGAAGTGGT
Tnf	CAACGCCCTCCTGGCCAACG	TCGGGGCAGCCTTGTCCCTT
